# Multidrug-Resistant* Escherichia coli* Resulting in Postpartum Necrotizing Endomyometritis

**DOI:** 10.1155/2019/6715974

**Published:** 2019-04-17

**Authors:** Joan Tymon-Rosario, Meleen Chuang

**Affiliations:** ^1^Montefiore Medical Center, Department of Obstetrics & Gynecology and Women's Health, Bronx, NY, USA; ^2^Albert Einstein College of Medicine, Bronx, NY, USA

## Abstract

**Background:**

Postpartum endometritis is a fairly common postoperative complication occurring in up to 11 percent of all cesarean deliveries. Multidrug-resistant pathogenic organism is increasingly a factor in postoperative source of infection. Postpartum endomyometritis from a multidrug-resistant* Escherichia coli* infection resulting in uterine is one such rare clinical circumstance where there is minimal information in the literature to guide its treatment and management.

**Case:**

A 29-year-old G1P0 who underwent a primary cesarean delivery for a failed induction of labor developed endomyometritis on post-op day one and was treated with multiple broad-spectrum antibiotic regimens. The source of infection was found to be multidrug-resistant* Escherichia coli *with uterine involvement and pelvic abscesses, requiring hysterectomy and drainage of pelvic abscesses. Severe uterine necrosis from this multidrug-resistant* Escherichia coli *infection was noted intraoperatively. After three weeks of antibiotic therapy, she had resolution of her infection.

**Conclusion:**

Multidrug-resistant* Escherichia coli *is a highly pathogenic organism that can cause endomyometritis, persistent bacteremia, and uterine necrosis, which necessitates definitive surgical management with hysterectomy to achieve resolution of the infection.

## 1. Introduction

Postpartum endometritis is an infection of the decidua that may extend to the myometrium resulting in endomyometritis. The most important risk factor for the development of postpartum endometritis is cesarean delivery after onset of labor. The incidence of endometritis is approximately 6 percent for primary cesarean deliveries performed before labor and up to 11 percent for deliveries performed during labor [1]. The infection is typically polymicrobial involving a mixture of two to three anaerobes and aerobes form the lower genital tract. Early-onset infection and high fever are traditionally thought to be due to organisms such as group A or B beta-hemolytic* Streptococcus* [2]. Multidrug resistant organisms pose an increasing problem for patients resulting in septicemia and potential death. We report a unique clinical circumstance of a patient with endomyometritis from multidrug resistant* Escherichia coli* resulting in uterine necrosis.

## 2. Case Description/Summary

A healthy 29-year-old G1P0 at 39w5d was admitted for labor induction secondary to decreased fetal movement and indeterminate fetal heart rate tracing. She has no past medical (significant for baseline anemia (hemoglobin of 9.0) during pregnancy) or surgical history. Her labor induction involved a single dosage of 25 mcg of Misoprostol per vagina followed by cervical Foley insertion with Oxytocin administration for approximately 30 hours. She underwent a primary cesarean delivery for Category II fetal heart rate tracing and arrest of dilation at 5 centimeters. The cesarean delivery was performed without complication.

On postoperative day one, the patient was febrile (38.8°C), hypotensive (80-95/40-55), and tachycardic (120-140). The patient was diagnosed with sepsis from endomyometritis and was started on intravenous ampicillin, gentamycin, and clindamycin. Sepsis workup included blood and urine cultures, laboratory studies, and chest x-ray. Laboratory studies indicated hemoglobin of 7.0 and she underwent a transfusion of two units of packed red blood cells with an appropriate rise to hemoglobin of 9.3. On postoperative day two the patient was hemodynamically stable but still remained febrile (T Max 39.3°C). The urine culture and blood cultures were positive for* Escherichia coli. *Infectious disease (ID) consultation recommended a new antibiotic regimen of intravenous piperacillin-tazobactam. Sensitivities of the organism demonstrated a multidrug resistant (MDR)* Escherichia coli* and the regimen was changed to intravenous meropenem. On postoperative day three, a transabdominal ultrasound showed no retained products of conception, a thin endometrial stripe, and no evidence of endometrial abscess. Computed Tomography (CT) scan of the Abdomen and Pelvis was performed later that day and demonstrated a 2.6 x 2.5 cm defect by the cesarean delivery hysterotomy below the fascia with fluid, small amount of complex abdominopelvic ascites with few gas bubbles. No urinary tract pathology was evident on imaging.

**Figure 1:**
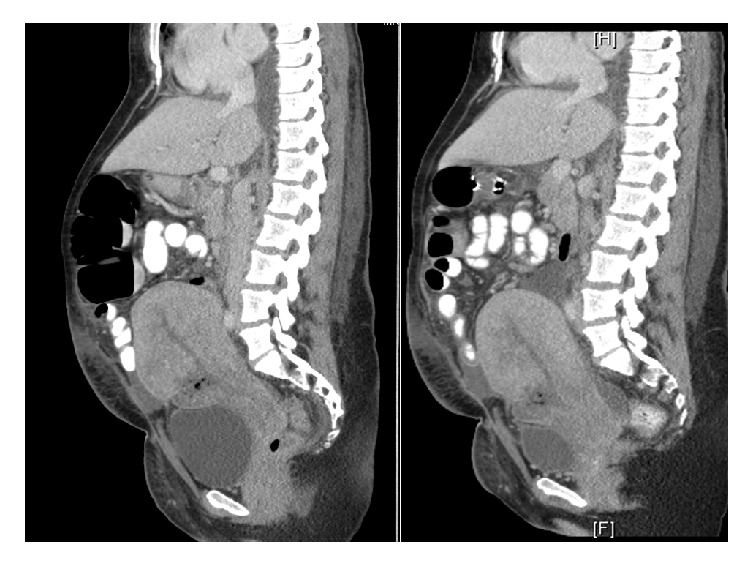
On the left, there is a CT A/P with IV contrast in sagittal view demonstrating that the uterus is enlarged and heterogeneously consistent and an approximately 2.6 x 2.5 cm defect is noted at the C-section incision site containing hypodense fluid and multiple bubbles of gas. Also, a focus of gas is also noted within the endometrial cavity. On the right, there is a CT A/P with IV contrast in sagittal view done two days after the one on the left demonstrating a persistent 2.6 cm defect at the C-section incision site containing fluid and multiple bubbles of gas. Now this defect is continuous with a 3.3 x 0.9 cm abscess along the anterior aspect of the uterus.

ID recommended additional oral metronidazole and heparin therapeutic anticoagulation for concerns of septic pelvic thrombophlebitis. The patient continued to be febrile the next 24 hours and repeat CT imaging demonstrated the 2.6 cm wound defect with a new abscess measuring 3.3 x 0.9 along the anterior aspect of the uterus and in the rectouterine space (Figure 1). The uterine endometrium appeared heterogeneous with one foci of gas. The oral metronidazole was discontinued and vancomycin was started. The patient continued to have fevers for the next 48 hours and was counseled for hysterectomy. The patient consented to the procedure and underwent an exploratory laparotomy, abdominal washout, total abdominal hysterectomy, bilateral salpingectomy, and Jackson Pratt drain placement on postoperative day nine after her initial primary cesarean section. Intraoperatively, there was purulent ascites with a severely necrotic uterus.

After the hysterectomy, the patient remained on meropenem and vancomycin. Cultures obtained from surgery grew primarily* Escherichia coli* and* Enterococcus faecalis. *After the hysterectomy, the bacteremia resolved, but fevers continued through postoperative day five after hysterectomy. CT imaging performed showed two pelvic abscesses that were ultrasound-guided drained with pigtail placement. The patient remained afebrile with several consecutive negative blood cultures. The drain was removed and the patient was discharged on postoperative day eleven on Bactrim and Augmentin for one week. At her postoperative check, she was doing well and at her six-week visit she had healed completely.

## 3. Discussion

Endomyometritis is common diagnosis among cesarean deliveries after labor/ruptured amniotic membranes. In this case, the severity of MDR* Escherichia coli *infection leads to uterine necrosis and requiring hysterectomy of healthy young women makes this case interesting. Multiple authors have described cases of uterine necrosis related to intrapartum or postpartum complications that increased the risk of infection. The reports in literature are of uterine necrosis after placement of B-Lynch compression suture, uterine artery arterial embolization, or surgical ligation techniques during management of postpartum hemorrhage. These procedures may be risk factors for infection as the tissue is devascularized. The only case similar to our case was described by Nigam et al., in which there was delayed myometrial necrosis after a primary cesarean delivery of a full term fetal demise with intra-amniotic infection [2]. Rivlin et al. described uterine necrosis after cesarean delivery from group B beta-hemolytic* Streptococcus* [3].

There are several established reports of group A or B beta-hemolytic* Streptococcus* organisms causing postpartum necrotizing myometritis [3–5]. MDR* Escherichia coli *infection is usually associated with urinary tract related infections and bacteremia [6–9]. This case shows how MDR* Escherichia coli* can cause severe postcesarean infections and necrotizing endomyometritis.

We acknowledge this is a single case report of a personalized treatment plan, which limits the application of our findings to other patients. However, this case is the first to report MDR* Escherichia coli* as a cause of uterine necrosis of a healthy patient without any risk factors that would place her at risk for such a severe infection. She was without any prior medical problems (nondiabetic, nonobese, nonsmoker, and not immunocompromised) and underwent uncomplicated cesarean delivery that did not require uterine artery embolization or a compression suture. This case highlights the morbidity of MDR* Escherichia coli* as a cause of uterine necrosis.

## Additional Points

*Precis*. To present a case in which endomyometritis from multidrug resistant* Escherichia coli* resulted in bacteremia and uterine necrosis necessitating definitive surgical management with hysterectomy.* Teaching Points*. (1) Multidrug resistant* Escherichia coli *is a rare, highly pathogenic organism that can cause endomyometritis and may result in postpartum uterine necrosis. (2) Postcesarean endomyometritis is treated with broad spectrum antibiotics. Persistence of bacteremia may indicate multidrug resistant organisms and workup should reflect this. One must consider hysterectomy as last resort to remove source of infection. (3) Quality improvement goals should focus on the reduction of hospital acquired infections; as these highly pathogenic and antibiotic resistant organisms can cause severe infections.
